# Supplementary material for: Comparative multiomics analysis of cell physiological state after culture in a basket bioreactor
Source: Sci Rep. 2022 Nov 23;12:20161. doi: 10.1038/s41598-022-24687-4 (PMC9686226; doi:10.1038/s41598-022-24687-4)
Supplement: Supplementary file 1 — Supplementary Information 1. [file 41598_2022_24687_MOESM1_ESM.zip › raw data/Transcriptomic raw data/6.Enrichment/1.GO/TE1vsTC1/ALL/TE1vsTC1.all_GObar_graph_updown.pdf]

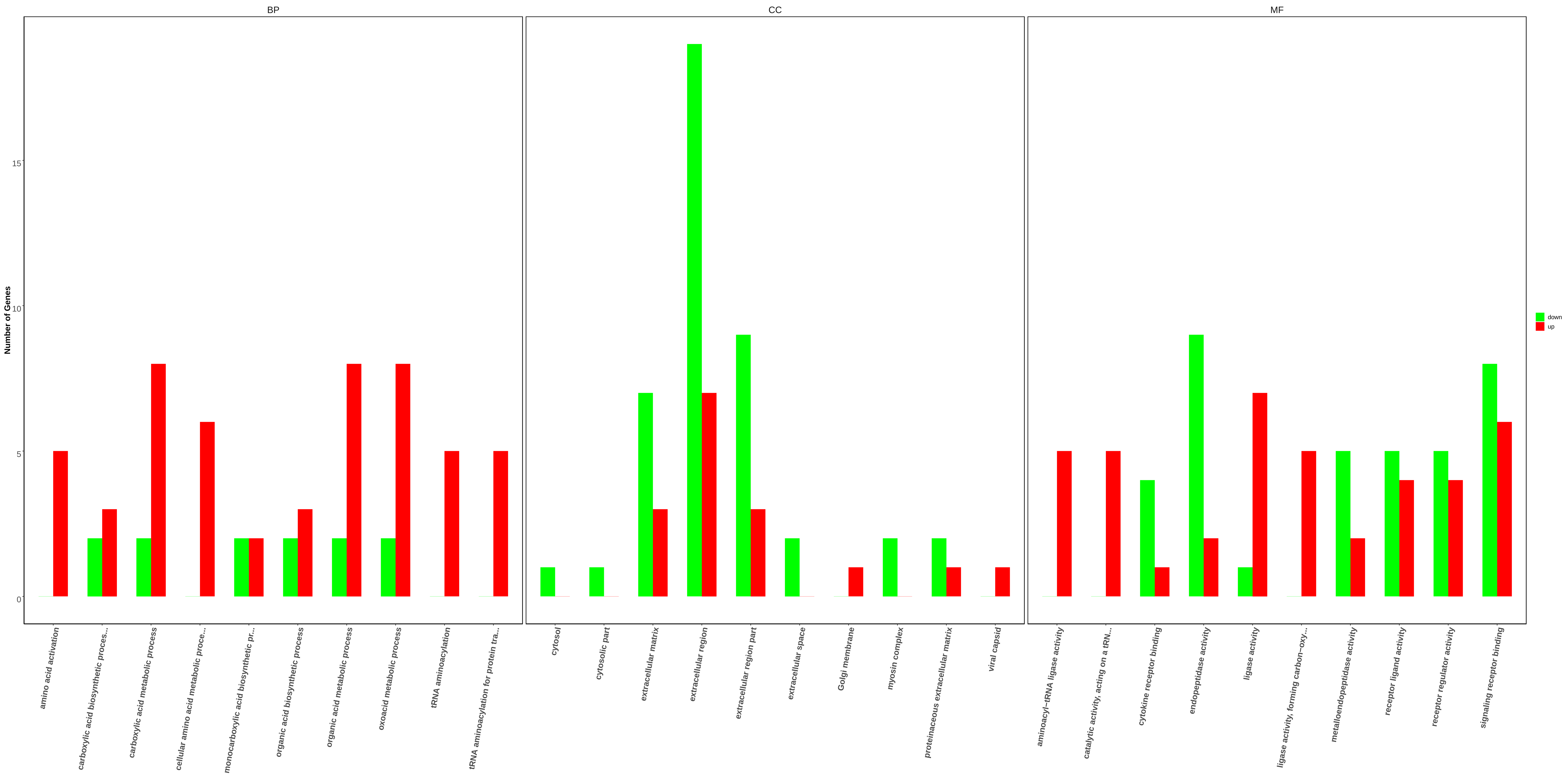
BP
CC
MF
Number of Genes
0
5
10
15
down
up
amino acid activation
carboxylic acid biosynthetic proces...
carboxylic acid metabolic process
cellular amino acid metabolic proce...
monocarboxylic acid biosynthetic pr...
organic acid biosynthetic process
organic acid metabolic process
oxoacid metabolic process
tRNA aminoacylation
tRNA aminoacylation for protein tra...
cytosol
cytosolic part
extracellular matrix
extracellular region
extracellular region part
extracellular space
Golgi membrane
myosin complex
proteinaceous extracellular matrix
viral capsid
aminoacyl−tRNA ligase activity
catalytic activity, acting on a tRN...
cytokine receptor binding
endopeptidase activity
ligase activity
ligase activity, forming carbon−oxy...
metalloendopeptidase activity
receptor ligand activity
receptor regulator activity
signaling receptor binding
